# Who is responsible for assessing children’s weight status? – a qualitative study of health professionals in regional Australia

**DOI:** 10.1186/s12889-019-7539-x

**Published:** 2019-08-30

**Authors:** Kamila Davidson, Helen Vidgen, Elizabeth Denney-Wilson, Lynne Daniels

**Affiliations:** 1 School of Exercise and Nutrition Sciences, Faculty of Health, Queensland University of Technology, Kelvin Grove Campus, O Block, A Wing, Victoria Park Road, Kelvin Grove, Brisbane, Queensland 4059 Australia; 2 Sydney Nursing School, The Universtiy of Sydney, 88 Mallett St, Sydney, New South Wales 2050 Australia

**Keywords:** Children, Guidelines, Health professionals, Obesity, Overweight, Primary health care, Weight status assessment

## Abstract

**Background:**

Currently in Australia there is a lack of clarity regarding routine assessment of primary school aged children’s weight status despite it being the first step in the identification of overweight and obesity. The National Health and Medical Research Council Obesity Guidelines recommend primary health care professionals include routine weight status assessment in consultations with children yet research suggests this rarely occurs in practice.

This study aimed to determine the views of primary health care professionals regarding routine weight status assessment in primary school aged children and to establish the barriers to assessing children’s weight status.

**Methods:**

Using the case study of a regional town, Rockhampton, purposeful sampling was used to represent the key primary health care settings and professional groups. Interviews were conducted with 31 health professionals. Data were collected and analysed guided by two frameworks, the Capability, Opportunity, Motivation and Behaviour and Theoretical Domains Frameworks.

**Results:**

Eight themes emerged from data and these were relevant to the three levels of influence on the routine weight status of assessment, system, setting and individual. System level themes related to having a formalised program for the undertaking of routine weight status assessment in primary school aged children, increasing the population’s awareness about the importance of the weight status check and limited public health services available for management of childhood overweight and obesity. Setting level theme regarded the location where routine weight status in primary school aged children could be undertaken. Four themes at the individual level of influence on the routine weight status assessment related to the primary health professionals’ roles, barriers to assessing children’s weight status, methods of weight status assessment and starting a weight related conversations with families.

**Conclusion:**

The Government, primary health care services, professional organisations and associations as well as health professionals must commit to long-term implementation of the Obesity Guidelines. Immediate action to improve the undertaking of routine weight status assessment in children must be taken by each health service and health professional. Strategies should aim to positively affect motivation to assess children’s weight status as it is the central component in creating change in practice.

## Background

Childhood overweight and obesity can be identified through a number of assessments, some of which can be in-depth and burdensome on the families, health professionals and the health care system. In Australia, the National Health and Medical Research Council (NHMRC) “Clinical Practice Guidelines for the Management of Overweight and Obesity in Adults, Adolescent and Children in Australia (Obesity Guidelines)” [1] recommend to follow the “5As” framework (ask and assess, advise, assist and arrange) when managing overweight and obesity. Prior to progressing into detailed investigations health professionals should focus on completing the “ask and assess” step, which includes routine assessment of children’s weight status [1]. No formal incentives are available for the undertaking of this check. Instead, it is recommended to incorporate this assessment, into standard care, of children and use objective methods to undertake it, namely gender- and age- specific Body Mass Index (BMI) percentiles [1]. Once the assessment is completed health professionals are to discuss results with parents and then advise them on what can be done to maintain child’s healthy weight status or address overweight or obesity [1]. Whilst weight status assessment is often an element of a formalised early years developmental health checks [2, 3], once children commence school their contact with health settings for health checks decreases and, despite the Obesity Guidelines’ recommendations, their weight status is rarely assessed in standard consultations [4–7]. Australia, unlike some countries [8], does not offer this assessment on a regular basis as a mandated program for primary school aged children. This is why the undertaking of this health check is particularly important in this age group in Australia as it is the only opportunity to identify any weight related issues by a health professional and commence management. In spite of this, it is unknown who is responsible for implementation of the “ask and assess” step of the Obesity Guidelines [1].

It is recommended that routine weight assessment of children is undertaken at the primary level of the health care system [1]. Primary health care is the first point of contact between the public and the health system and internationally is recognised as “essential health care” [9]. In Australia, primary health care is said to be “the frontline of Australian health care system” and offers a range of services via the public, private and non-government sectors [10]. This puts primary health care and health professionals working within its settings in a key position to undertake routine health checks, such as weight status assessments, identify any growth issues early and manage them accordingly. Australian primary health care settings can be broadly divided into three models: general practice, general community-based care and other settings (for example ambulant or Indigenous-specific services) [11]. A wide range of health professionals are employed across these models and are the potential implementers of the Obesity Guidelines [1]. In addition, the range of primary health care services available to the public may differ depending on the geographical location and the local population’s needs. Therefore it may be unclear to the public and health professionals who is or should be assessing children’s weight status. This may result in the absence of action with consequences for the child, family and other health professionals.

While a range of health professionals interact with families in health care settings, Australians most commonly consult general practitioners (GPs) [12]. General practitioners and the general practice setting has therefore been the focus of a number of previous studies investigating children’s weight status assessment and management of childhood overweight and obesity [5, 6]. The results highlight that GPs, in Australia [5–7] and in other countries [13, 14], do not routinely assess children’s weight status as a part of standard care during appointments despite reportedly feeling confident and competent in assessing weight status and communicating the results to parents [5, 6]. Australian studies reported that as little as 3 and 9% of GPs (*n* = 34 and 85 respectively) assessed BMI of children aged 5–10 and 2–10 years, respectively [5, 7]. Thus it seems unlikely that further research focusing solely on GPs’ implementation of the Obesity Guidelines [1] will create any change in practice unless there is clarity about who is responsible for routine weight status assessment of children and the current barriers to undertaking of this check are thoroughly investigated and addressed. Although the prevalence of childhood overweight and obesity is high [15], weight related concerns are unlikely to be the main reason for the family’s visit to a GP [4, 16]. This suggests that other health professionals, who also operate in the “frontline” health services, might be well suited to routinely undertake this health check. It is currently unknown who primary health care professionals see as best positioned and equipped to assess children’s weight status and raise concerns about overweight and obesity with parents, and where this health check could be routinely undertaken. Therefore, the aims of this study are to (1) determine the views of health professionals, who are the intended users of the Obesity Guidelines [1] and work in a range of primary health care settings, on routine weight status assessment for primary school aged children; and (2) to explore the barriers to assessing primary school aged children’s weight status. This study forms a part of a project which explored routine assessment of children’s weight status and the responsibility for its undertaking. It was undertaken alongside a quantitative study which focused on parental opinions about routine undertaking of children’s weight status [17].

## Methods

### Design

This study undertook a case study approach and focused on a regional city of Rockhampton in the State of Queensland, Australia. The reasons for selecting this area included its location, obesity prevalence and availability of health services, and ability to discuss these reasons with local stakeholders to determine if there was a need for research on this topic in the area.

Rockhampton is a city of almost 80,000 people [18] located approximately 600 km from Brisbane, the State’s capital city. Adult obesity in the area of interest was at 29% (2011–12), which was 6% higher than the State’s prevalence [19] (childhood obesity rates are unavailable for this area however it was at 7.2% for children aged 5–17 years old in Queensland in 2014–15 [20]). Rockhampton provides health services at the primary, secondary and tertiary level, and offers services representing each of the three models of primary health care. While metropolitan areas may offers larger choice of and accessibility to a number of health care services, both public and private, this adds to the complexity of who is or could be responsible for assessing children’s weight status. On the other hand, rural or small regional areas are unlikely to offer health services across primary, secondary and tertiary level of health care, or services from a range of health professionals across the three models of primary health care. With fewer health services available in regional towns such as Rockhampton there are less opportunities for competition or repetition which may be the case in larger cities. There is also potential for greater communication and collaboration among health services in regional areas as they work towards providing comprehensive health care. Further, due to Rockhampton’s location its residents mostly, if not entirely, rely on health services available in the area and it is unlikely that families would travel outside of this town for their children’s routine health checks or management of overweight and obesity. Consultations with Rockhampton-based health professionals confirmed assumptions made about this area and ascertained there was a need for local research on childhood obesity management.

### Sample recruitment

This study aimed to recruit health professionals who operate in the health services across three models of primary health care. Purposeful sampling strategies were employed including maximum variation (in setting, service and profession) and snowball (chain), to recruit a diverse and information-rich sample.

**Fig. 1:**
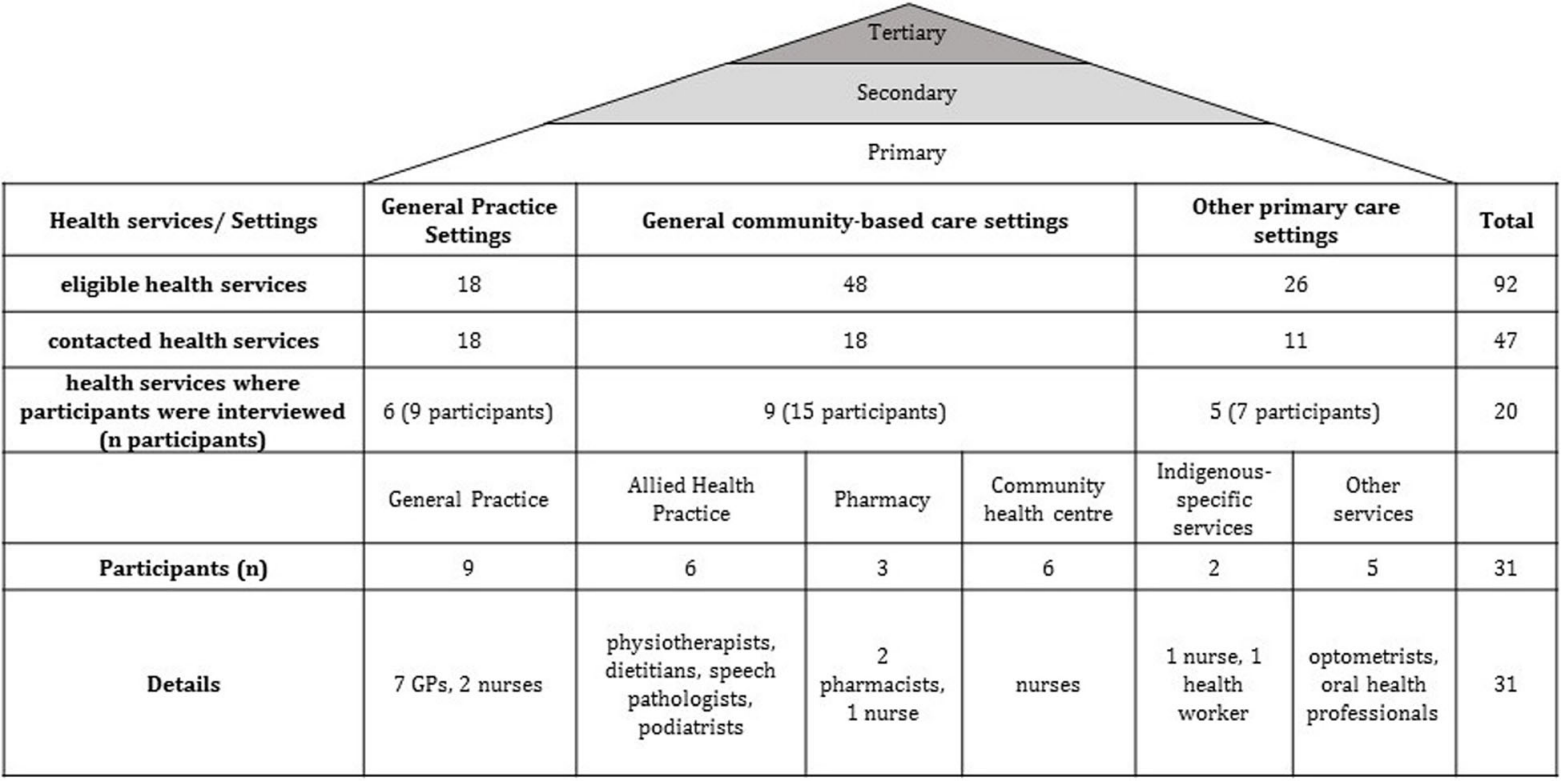
Health care levels, primary health care setting, recruitment process and participants

A list of 92 Rockhampton-based primary health care services, which offered services to children, was developed, based on searches using the National Health Service Directory [21]. This study was also promoted via the authors’ existing professional network groups in Rockhampton and included contacts in the Public Hospital and Community Health Clinics. The eligibility criteria required the potential participants to operate in a primary health care service, and interact with children as a part of their professional role. Recruitment and data collection occurred simultaneously. The results determined where further recruitment was to take place to ensure representatives from different health care settings, services and professions were included in the sample. Out of the 92 services that were identified, 47 were progressively contacted via email, telephone or in person and invited to participate. Recruitment and data collection continued until data saturation was reached, which is why some health services were not contacted. Twenty different primary health care services representing all three models of primary health care were engaged and a total of 31 health professionals were interviewed. Figure 1 outlines where the sample was recruited from, setting, health service and study participants.

### Interview guide

**Fig. 2:**
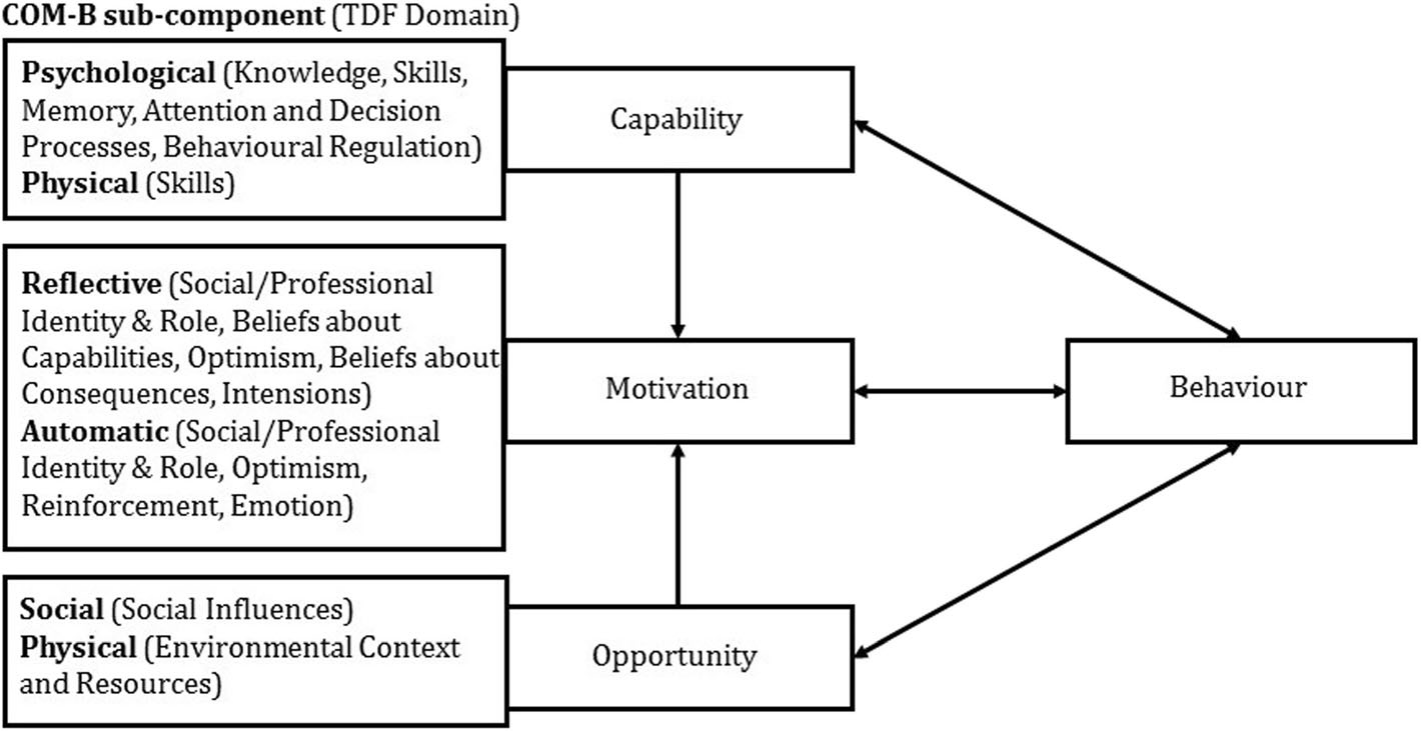
The COM-B system, its subcomponents and corresponding TDF domains [22, 24]

Interview questions and the corresponding prompts were based on the Capability, Opportunity, Motivation, Behaviour (COM-B) system and the Theoretical Domains Framework (TDF). The COM-B system, a simple but comprehensive model for understanding behaviour, has three components, Capability (C), Opportunity (O) and Motivation (M), which impact one another and influence Behaviour (B) (Fig. 2) [22]. The TDF allows for in-depth exploration of the COM-B system’s sub-components and combines a number of theoretical constructs into one framework which comprises fourteen domains and these correspond to the components/sub-components of the COM-B system [23]. Whilst the TDF can be applied without the COM-B system, it is recommended to use the COM-B system when behaviour of interest is not well understood [24] which in the case of this study is routine weight status assessment of primary school aged children.

Questions were piloted for face validity with a convenience sample of three health professionals who live outside of Rockhampton area. Interview questions examined opinions about routine assessment of primary school aged children according to the three components of the COM-B system and its corresponding TDF constructs.

- Who is seen as having/who could have the **Capability** to routinely assess primary school aged children’s weight status?
  Example questions include:
  - Who has the skills and knowledge of how to assess?
  - Who would have learnt how to assess and interpret the results?
  - Who would be best equipped to discuss the results with parents?
  - How do you feel about undertaking of this assessment and discussing the results with families?
- Who is seen as having/who and where could have the **Opportunity** to routinely assess primary school aged children’s weight status?
  Example questions include:
  - Where do you see the opportunity to assess the weight status?
  - Who has the necessary equipment?
  - What type of system, if any, do you use?
  - How might views/opinions of others affect decision to assess children’s weight status?
- Who is seen as having the **Motivation** to routinely assess primary school aged children’s weight status?
  Example questions include:
  - Who do you think has a role in assessing children’s weight status?
  - How does it fit within your/other health professionals’ role?
  - Why do you undertake this assessment?
  - How easy/difficult do you find it to assess weight status and discuss the results with families?
  - How do you feel about undertaking of this health check?
  - Do you think it is worthwhile doing?

Interviews took place in June–July 2016, lasted on average 14 min and were undertaken by the first author either face-to-face or via telephone. All interviews were audio recorded and transcribed verbatim prior to analysis. Participants were offered the option to review their transcripts but all declined. Ethics approval was obtained from the Queensland University of Technology (number: 1600000512).

### Analysis

Data were de-identified and coded according the theoretical components of the COM-B and TDF with the use of NVivo 11 software [25]. A four-step analysis was undertaken. (1) The first two authors coded data according to COM-B components/sub-components and its corresponding TDF constructs. The first author coded all transcripts and the second author independently coded four transcripts. The first two authors discussed the coding discrepancies and resolved disagreements. Some data were coded under two theoretical constructs, for example the same evidence was coded under Social/ Professional Identify and Role for Motivation/ Reflective and Motivation/ Automatic. There were no data coded under Motivation/Automatic/Optimism (2). As similar codes were given to evidence across COM-B components/sub-components and its corresponding TDF constructs these were grouped together by the first author into eight themes (3). The eight themes were divided according to their relevance to one of the three levels of influence on routine weight status of assessment, system, setting and individual. System level influences were those relevant to the government policies or legislation, availability of health services as well as population-wide interventions or campaigns. Setting level influences related to each health service and the setting it operated in, for example, suitability of a health setting for the undertaking of routine weight status assessment. Individual level influences include those affecting an individual health professional and the professional discipline, for example, GPs, nurses or allied health professionals (4). The COM-B components/sub-components and their corresponding TDF constructs were linked with intervention functions in the Behaviour Change Wheel (BCW).

The BCW is a tool for designing behaviour change interventions and is directly related to the COM-B and TDF, as these frameworks sit within the centre of the wheel, which is the behaviour of interest [22, 24]. Once influences on a behaviour are identified via COM-B and TDF these can be applied to the BCW which then allows for providing specific recommendations for intervention functions and policy categories aiming to achieve desired behaviour change [22, 24]. These should be appraised according to their affordability, practicability, effectiveness and cost-effectiveness, acceptability, side-effects/safety and equity (the APEASE; described in detail elsewhere [24]) which then allows to recommend Behavioural Change Techniques (BCTs) to develop strategies for behaviour change.

## Results

Interviews were undertaken with health professionals from a range of disciplines who worked in various health services across the primary health care. Most GPs and nurses reported having regular contact with primary school children whilst allied health professionals, pharmacists and other health professionals had limited interactions with children at the age of interest. As all were the intended users of the Obesity Guidelines [1] they therefore shared their perspectives on routine assessment of weight status of primary school aged children and provided views on how the undertaking of this health check could be implemented within the current health care system.

Eight themes across three levels of influence on routine weight status assessment of primary school aged children were identified from the interview transcripts and these together with their corresponding COM-B components/sub-components and TDF constructs are presented below and in Table 1. The themes are discussed under their relevant level of influence on routine weight status assessment.

**Table 1:** Themes mapped against level of influence, the COM-B and TDF frameworks’ constructs

Level of influence	Theme number	Theme	COM-B component/COM-B subcomponent (TDF domain)
System	1	System approach to weight status assessment is required to normalise the undertaking of this check	Capability/Psychological (Behavioural Regulation), Opportunity/ Social (Social Influences), Motivation/ Automatic (Reinforcements)
	2	Increasing public awareness about the importance of weight status assessment	Capability/ Physiological (Behavioural Regulations), Opportunity/ Social (Social Influences)
	3	Limited public health services available for management of childhood overweight and obesity	Capability/ Psychological (Skills), Capability/Physical (Skills) Opportunity/ Physical (Environmental Context and Resources)
Setting	4	Schools versus other settings were weight status assessment could be undertaken	Opportunity/ Physical (Environmental Context and Resources)
Individual	5	Recognition of GPs’ and nurses’ role in children’s routine weight status assessment	Capability/Psychological (Knowledge), Capability/ Psychological (Skills), Capability/Physical (Skills), Motivation/ Reflective and Automatic (Social/Professional Identity & Role), Motivation/ Reflective (Beliefs about capabilities)
	6	Barriers to assessing weight status in primary school children and discussing the results with families	Capability/ Psychological (Memory, Attention and Decision Processes), Motivation/ Reflective (Beliefs about Capabilities), Motivation/ Automatic (Emotions)
	7	Objective and subjective methods of weight status assessment	Capability/Psychological (Memory, Attention and Decision Processes), Motivation/ Reflective (Beliefs about Capabilities), Motivation/ Reflective (Beliefs about consequences)
	8	Conditional raising of weight status topic with parents	Capability/ Psychological (Memory, Attention and Decision Processes), Opportunity/ Social (Influences Social), Motivation/ Reflective (Beliefs about Consequences), Motivation/ Reflective Intentions (Motivation and Goals)

### System level

There were three themes identified in the data that can be considered under the system level of influence on the routine weight status assessment. These relate to having a formalised program for the undertaking of routine weight status assessment in primary school aged children (theme 1), increasing the population’s awareness about the importance of this check (theme 2) and limited public health services available for management of childhood overweight and obesity (theme 3).

#### Theme 1: system approach to weight system assessment is required to normalise the undertaking of this check

The majority of health professionals perceived the lack of a system as a barrier to having this health check done, and that having a standardised system with well-trained operators, not necessarily health professionals, may improve routine undertaking of children’s weight status.

*“Unless you have a system, nothing happens. It’s like with other rules, unless you legislate nothing happens.” (General practice setting/GP#3)*

Some health professionals commented that although a system for weight status assessment or reminders sent to parents may be useful, the challenges identified by the participants to set such system up may out way potential benefits. Some of the recognised difficulties were communication with parents (consent and child’s baseline medical history provision, referrals and follow-up), employment of implementation staff (availability and appropriate qualification and training of staff, acceptability from the perspective of school, parents and children), collection and handling of sensitive data and risk of bullying among children. However, many health professionals expressed a belief that once a system or program for weight status assessment is established and runs for a period of time having children’s weight status checked and reported on will become a standard procedure.

Some participants reflected on whether receiving additional rebates from the national health insurer, Medicare, would encourage health professionals in a general practice setting to routinely assess children’s weight status. Opinions about introducing incentives for the undertaking of this check, as a part of formalised system or as a part of standard consultation, were mixed. One GP commented that additional rebates might encourage routine weight status assessment in primary school aged children.

*“If the government creates a Medicare rebate for something like that, it means that there's huge number through the door because it's income.” (General practice setting/GP#2)*

Another GP appeared to feel strongly against introducing Medicare rebates due to a belief that it is something that GPs are already taught and required to do as a part of standard care.

*“I’m doing it [routinely assess weight status in children]. There's no Medicare incentive. I'm doing it simply because it is very important to monitor the child's, as I said, growth and development. And for me, that's my practice. That's part of what they teach us. I don't know what the other GPs are doing but I certainly have been doing it.” (General practice setting/GP#4)*

#### Theme 2: increasing public awareness about the importance of weight status assessment

A few health professionals commented on the need to increase public awareness about the importance of routine weight status assessment in childhood in the community and in medical practices. The participants recommended running a public health campaign and having more promotional materials (posters, leaflets) in waiting rooms in doctors’ practices. One nurse commented on the lack of awareness and available resources and the need for these in practices by saying:

*“There’s nothing promoting to GP centres, like, there’s nothing saying: have you done this? […] there’s nothing to act on as the nurse to assess these. If there’s more and it’s in a general practice then yes we’d probably be pushing it [assessing weight status] more. If there is more awareness from schools like with the speech and stuff. They’d be probably pushing it more but there is no awareness for parents to GPs to monitor it. […]. There needs to be a more awareness for that to happen.” (General practice setting/Nurse#1)*

#### Theme 3: limited public health services available for management of childhood overweight and obesity

One of the barriers to starting weight related conversations with families was the recognised lack of public health services for childhood overweight and obesity management. Many health professionals commented on having little to no free and universally available services to which they could refer Rockhampton families or children requiring intervention. If overweight or obesity was suspected or identified, health professionals said that they usually refer (or would refer) the family to their GP or a private allied health professional (exercise psychologist or dietitian). Referrals to private health services where rare due to their cost and limited choice of paediatric health services in the area.

*“Funding doesn't reach regional areas a lot of the time. We tend to see a lot of programs that are run in areas down towards Brisbane where they have huge numbers of staff on projects because that's their trial sites and then, often it doesn't roll on to other areas which is a shame.” (Community health centre/Nurse#2)*

### Settings level

There was one setting level theme identified and related to the location where routine weight status in primary school aged children could be undertaken (theme 4).

#### Theme 4: schools versus other settings were weight status assessment could be undertaken

Health professionals discussed two main settings where weight status could be assessed: schools and general practices. Due to easy access to all children, schools were identified as most convenient and feasible setting for weight status assessment from a logistics perspective. Most GPs and nurses thought that school would be a suitable setting for this health check providing that well-trained staff are provided. Some health professionals however expressed concerns about weighing children in the absence of their parents and possible increased risk of bullying.

General practices were believed to be the most acceptable settings where children’s weight status could be assessed due to parental presence at appointments and a conceptual connection patients can usually make between GPs or nurses and health checks. However, health professionals recognised a number of challenges associated with having a weight status assessment undertaken in a general practice in the current health care system. These challenges included limited access to children, time constraints and focus on treating illness rather than prevention of disease.

Whilst other primary health care settings, such as pharmacies, optometry clinics, dental services, allied health clinics, were discussed by some of the participants as potential settings for weight status assessment these were mostly seen as inappropriate. A number of reasons were cited by the participants and included limited relevance of scope of practice of the individual health professional with addressing overweight and obesity, lack of capacity within the time of consultations or interaction with the family and the public seeing undertaking of this check in these settings as irrelevant to their appointment.

### Individual level

Four of the themes identified from the data were relevant to the individual level of influence on the routine weight status assessment in primary school aged children. These themes related to the primary health professionals’ roles (theme 5), barriers to assessing children’s weight status (theme 6), methods of weight status assessment (theme 7) and starting a weight related conversations with families (theme 8).

#### Theme 5: recognition of GPs’ and nurses’ role in children’s routine weight status assessment

Overall, GPs were recognised by the participants as gatekeepers of child’s health information and the first point of referral when other health professionals have any concerns regarding children’s health. While the undertaking of weight status assessment was considered a task which does not require a health professional it was recognised that a health professional with extra training in child health area should be relaying the assessment’s results to parents. Health professionals recognised the capability of GPs’ and nurses’ to conduct weight checks and discuss the implications. While GPs and nurses saw their own role to include undertaking of the weight status, many of them reported having little to no capacity or interest in doing so. This was expressed when providing reasons or barriers for not including this check in the standard consultation. Some GPs identified lack of specific training in children’s weight status assessment, and not being taught or told to complete this health check during a standard consultation.

*“It’s never been trained into us as a thing that you should always do. Unlike adults with blood pressure or whatnot, you always think about opportunistic testing and things like that, not so much with weight, with kids, it's always about, “Right, I’m going to start them on some medication. I need to dose them appropriately so I’d weigh them”. And even that, unless it is dramatically out of normal, we don’t even look at the charts and compare and see what the percentile they are in, the general population, anyway. So in that sense, no, not really ingrained into us that we routinely test for it. And even if we do test for it, it’s with the idea of dosing medications, not so much the idea of ‘how’s their weight been going” (General practice setting/GP#6)*

The majority of health professionals other than GPs and nurses did not see weight status assessment as a part of their professional scope of practice. These participants reported that conversations about child’s weight are usually difficult for them to tie in with the reasons for presentation to their practice. Some health professionals also expressed concerns regarding parents failing to see such connection, which could make them feel uncomfortable, targeted or watched. There was a concern among some health professionals that the public may see undertaking of this health check out of context of the consultation especially when this assessment was to be done by non-GP or non-nurse.

*“I mean taking a child’s BMI would be seen as like that, I mean you are looking at their mouth, why do you need to know their weight. They wouldn’t see it as relevant to what we are doing, why do we need to be so nosy. […]. I think there must be some sort of separation [of roles in health care] because if there was all transparent then they [patients] would begin to feel like that there is like a big brother kind of feel to the care.” (Other primary health care setting/service/Oral health professional#1)*

However, when weight status was raised as a potential concern, the participants usually provided basic advice and referred the child to their GP, an exercise physiologist or a dietitian, recognising however a lack of public allied health services for children’s weight management in the region. Some of the interviewed health professionals also recognised that they could potentially extend their role to providing basic nutritional and physical activity advice and refer to GP for management.

While community child health nurses do not currently consult primary school children in the region or Australia, due to lack of capacity within their framework and funding, most of the participants, including child health nurses themselves, recognised that routine assessing of children’s weight status could be their role. Child health nurses expressed being interested in expanding their role to encompass children in primary school if additional funds were provided to their department. Further, they saw the importance of building rapport with children and parents as essential, especially when sensitive data were collected and discussed.

#### Theme 6: barriers to assessing weight status in primary school children and discussing the results with families

There was a consensus among all participants that raising weight-related concerns with families, whether weight status is assessed or not, is done inconsistently due to a number of barriers. Participants’ feelings or emotions related to assessing children’s weight status appeared to stop them from undertaking this health check. Many of the interviewed health professionals were concerned about offending or embarrassing the family, patients getting angry, losing clientele or a good patient-professional relationship, or making parents too focused on child’s weight. Some of the GPs in this study commented that it is difficult to raise any concerns regarding child’s weight status when the family who attends the consultation visually appears to be overweight or obese. Feeling as if a conversation about child’s weight status will have little to no impact on improving child’s health outcomes was expressed as a practice challenge by some of the GPs, and in some cases it stopped them from initiating weight-related discussions.

*“If I tell parents that their children are overweight, a lot of them get quite upset and angry at me. If, God forbid, if I tell them that they are obese or morbidly obese, because suddenly you’re the baddy and somehow they find that accusing, but they had no gauge by which to try and say my child is overweight.” (General practice setting/GP#3)*

A different perspective on why health professionals may not assess primary school children was provided by a nurse, who consults children younger than four years of age. According to this participant, health professionals may assume that by the time child reaches primary school age their weight status would have been discussed with a health professional at some stage. This could then potentially stop health professionals from initiating a conversation about the child’s weight due to an assumption that weight issue was previously raised and ineffectively addressed or ignored by the family.

Overall among the participants, there seemed to be a resigned and passive attitude towards assessing child’s weight status and addressing childhood overweight and obesity. All of the interviewed health professionals appeared to expect a negative response from parents when the child’s weight status is assessed or discussed. However, the comments provided here were usually related to the context of weighing a visually large child who health professionals thought would be overweight or obese.

*“you think it might be embarrassing for the child or the parent but I think that’s easy enough to get over if you are confronted with the kid who is clearly overweight” (General practice setting/GP#5)*

*“Usually if I do see a regular customer with a child with a weight issue I think I would feel reluctant to approach the carer, to approach the mother. Basically, I think there's always bit of negative connotations. I would be worried I might be insulting the parent how they're bringing up their kid.” (Pharmacy setting/Pharmacist #1)*

Some participants thought that parents do not want to know about child’s overweight and obesity or fail to recognise that this health issue is a problem in the first place. One participant, a GP, commented on the lack of public comprehension of what a healthy weight is and how a healthy weight child should look. There appeared to be a lot of frustration associated with failure of parents to recognise their child’s overweight and obesity.

*“We have a generation or possibly even two generations now of people who do not have a good understanding of nutrition. They do not have a good understanding of appropriate weight issues or models, or things like that”.*

*“So I think we’re well behind the eighth ball trying to now re-educate two generations of people as to what is a healthy diet, what is a healthy activity levels? What’s the healthy body shape? All of those sort of things.” (General practice setting/GP#3)*

#### Theme 7: objective and subjective methods of weight status assessment

The participants recognised the importance of making decisions on weight management based on a series rather than singular measurement. Despite this however, all of the interviewed GPs appeared to recognise the importance of routine undertaking of children’s weight status yet only one reported taking their routine and objective measurements, and expressed being surprised that this health check is not done by all GPs. This participant cited the Royal Australian Collage of General Practitioners’ guidelines which recommend this assessment to be done by all GPs for all children.

*“I’m doing it simply because it’s very important to monitor the child’s growth and development. As for me that’s my practice. That’s part of what they teach us. The Royal Australian College of General Practitioners has always made a mention of getting measurements on kids so we do it. So you just got to follow the guidelines and they’ve been there for many years” (General practice setting/GP#4)*

Some of the participants questioned the necessity of using objective methods for routine weight status assessment (weighing children on scales, measuring height, calculating BMI, using Paediatric Growth Charts) to determine if a child is overweight or obese. One GP reported feeling confident that visual assessment is very accurate in assessing children’s weight status and queried if the objective methods would identify overweight and obesity more effectively than eye-balling the child’s body shape or size.

*“You look at a kid and you pretty quickly know whether there clearly overweight so I don’t routinely measure every child […]. Visual screenings is pretty good, isn’t it? I mean vast majority of obese people are fairly easy to pick in kids and adults but I mean when you get down to the fine detail you can probably look at more the mild to moderate obesity aren’t you. If someone’s very obese then [they] stand out […]. I suppose you would need to research […] to find out to what extent those kids who are overweight […] were actually identified anyway. […] Whether the [routine weight status] assessment [using objective methods] does add to the diagnosis right, you know. Many of the screening, the screening, does it actually identify more kids than are already identified [using eye-balling].” (General practice setting/GP#1)*

Two participants, allied health professionals, expressed doubts in the need for weighing children. There were concerns among the participants with regard to using BMI as a tool to assess children’s weight status, such as its limitations when used in individuals with high body muscle mass. Doubts were expressed about using Paediatric Growth Charts (BMI-for-age) especially when relaying the results to parents who may misinterpret the data if it is not explained correctly. One allied health professional feared that the risks (psychological harm to the child) of using objective methods of weight status assessment could outweigh potential benefits.

A few participants expressed their concerns over the evidence on the value of assessing children’s weight status and questioned its worth as well as the need for it. One allied health professional questioned the value of undertaking of children’s weight status checks and voiced concerned that by doing so things may get worse for children rather than improve.

*“We have to also ask is there anything, any value? Is there any value in having the checks? I mean the most recent paper that I saw published was saying that when parents know that their child is overweight, the child is more likely to gain more weight, than if the parents don't believe their child is overweight. (…) I found that quite horrifying that we’re actually by identifying these children, we may, in fact, be making the problem worse, that we actually don't know how to deal with overweight and obesity in children. We don't have a good program, good treatment methods and we do run the risks of making things worse.” (Allied Health Practice/ Allied health professional#1)*

One nurse reflected on the use of BMI in childhood by saying:

*“Weight alone won’t necessary tell us whether a child is obese and BMI is not perfect either. But we know that. But it’s just another tool in our kit basically.” (Community health centre/Nurse#4)*

#### Theme 8: conditional raising of weight status topic with parents

There appeared to be an overall belief among the participants that people/patients do not expect to have their primary school aged children’s weight status assessed during standards consultations with health professionals. The majority of the interviewed health professionals expressed little to no intention to assess primary school children’s weight status or raise any weight related concerns during standard consultations and generally focused on assisting children with the presenting concern. Most GPs in this study reported checking children’s weight status only when they felt it was relevant to their consultation; for example, to prescribe appropriate medication dosage; or when GPs or parents are concerned about a child’s weight.

Some of the interviewed allied health professionals mentioned that child’s weight status could be raised during a non-weight related consultation when it could be a potential contributor to whatever condition the child presented with (for example a sprained ankle). However they failed to see how the conversation could start when child’s appointment was for reasons they saw as directly unrelated to weight (for example a finger injury).

*“If they come in, let’s say they come in here as a patient, they are obese but they are here for a pain in their thumb, then if I talk to them that they are obese then they will think that I’m wasting their time as well.” (Allied Health Practice/ Allied health professional#2)*

Many participants recognised the value in routine weight status assessment of primary school aged children however raising any concerns was seen as worthwhile only when parents appeared to be receptive to receiving this health check’s results. Being receptive was understood by the interviewees as parent asking for any information related to child’s weight status, weight, diet, physical activity or when the reason for consultation could be somehow related to child’s weight status.

## Discussion

This qualitative study aimed to explore the perspectives of health professionals working in Australian primary health care settings on routine weight status assessment of primary school children with the application of a behaviour change framework. The use of the COM-B and the TDF frameworks allowed for in-depth exploration of the research question and data analysis, which consequently assisted with making recommendations for change in practice. These frameworks were previously applied in a variety of settings to explore and understand and address behaviour in health care [26–28]. For example, in Australia COM-B and TDF were used to determine barriers and enablers to delivery of Healthy Kids Check among GPs (*n* = 22) in focus groups [26], in Germany these frameworks were applied in a study which interviewed primary health care providers (*n* = 12) about the management of vertigo [28] and in United Kingdom researchers used COM-B and TDF to explore parental food portion behaviours in focus groups with caseworkers (*n* = 4) and parents (n = 22) [27]. In this study, application of these frameworks provided an opportunity to highlight practice challenges associated with an essential step in management of childhood overweight and obesity among various health professionals, health services and settings.

The health professionals in our sample mainly expressed negative or resigned attitudes towards routine assessment of primary school aged children’s weight status while discussing a number of barriers, and engaging in any weight related conversations with families under specific circumstances. While having limited public health services where families can be referred to for childhood obesity management was recognised as a barrier to starting weight related conversations health professionals reported referring, or that they would refer, families to their GP which highlights their key role in early identification of obesity. While standard practice of each health professional were not examined, health professionals other than GPs appeared to follow similar practice of referring to the child’s GP. Despite this, GPs seemed to fail to see themselves as an ideal resource not only to routinely assess weight status but also to manage child’s overweight and obesity. As per previous research [29–33], health professionals in this study reported being worried about families’ negative reaction to weighing their child and discussing any deviations, and the potential for these reactions to compromise patient-health professional relationship. These barriers are consistent with those identified in other studies [29–33] and suggest the need for further training in communicating sensitive information with patients. With the Australian childhood overweight and obesity rates being high [15] it is important to develop and provide health professionals recognised as the audience of the Obesity Guidelines [1] with a clear case management pathway. The first management step within which routine weight status assessment sits, “ask and assess” [1], ideally, should be incorporated in every health professionals’ standard practice however it may be more beneficial to decide which single setting and which specific health professional is responsible for its undertaking. Nevertheless, a clear direction and training must be provided, either by the government, health care settings or professional organisations, as to how to start the conversation that enables assessment, concerns to be raised and followed-up, as these were the barrier to assessing or raising weight as a possible concern in the first place.

Despite the undertaking of routine weight status assessment during standard consultation being recommended by national Obesity Guidelines [1] this practice may not be specified by health professionals’ own professional associations. This may therefore cause a conflict regarding the role some health professionals see themselves and others in assessing children’s weight status or raising concerns about the weight status. Obesity Guidelines [1] identify a comprehensive list of primary health care health professionals who are recommended to include weight status checks in their standard consultations and comprises of GPs, nurses, Aboriginal health workers, allied health professionals. Although among the interviewed health professionals GPs and nurses recognised their own role in assessing children’s weight status, similarly to previous studies among GPs [5–7, 13, 14], they often failed to use their position to undertake weight status check or raise any concerns about risk of overweight and obesity when consulting children. With no formalised structure, at the system or setting level, to support the implementation of Obesity Guidelines [1] health professionals can make a choice of whether to conduct additional assessment in their standard consultation or not. Consequently, the majority of participants in this study did not include weight status assessment in their appointments with children and many failed to recognise this practice as a part of their professional role.

For most of the participants who considered having a role in weight status assessment, its routine undertaking did not appear to be a high priority. Further, it seemed to be of a lesser importance if parents attending the consultation seemed unreceptive to receiving weight related information or their presentation could not be directly related to child’s weight status. This is concerning especially when considering the results from a study among Irish GPs (*n* = 393) and parents (*n* = 457) of primary school aged children [13]. In this 2014 study, GPs appeared to be reluctant to weigh children while the majority of parents found weight status checks as useful and felt mostly positive or neutral about this practice [13]. Similarity to Australia, Ireland has high childhood overweight and obesity rates [13] and no formalised system for routine weight status check for school aged children [8]. Therefore, Irish parents are also likely to rely on primary health care professionals to identify weight related issues. The results from the Irish and the current study highlight therefore that health professionals, such as GPs, may be missing valuable opportunity during standard consultation to assess the weight status and raise weight related issues. This is concerning especially if no clarity regarding the responsibility for routine weight status assessment is provided in the future. It could result in little to no change in practice and health professionals continuing to rely on “others”, other health professionals or families, to commence a conversation about child’s weight status, identify and manage childhood overweight and obesity.

Objective methods of weight status assessment were not used or valued by all of the interviewed health professionals leaving identification of positive weight status balance up to subjective methods such as eye-balling. Health professionals in this study were confident in the accuracy of their visual assessment of children’s body size and did not see the need to validate these with physical measurement. This is concerning due to previous research [34] strongly suggesting that GPs who visually assess child’s body size rarely identify the child’s actual weight status category and often misdiagnose overweight as healthy weight, and obese as overweight children. Consequently, reliance on eye-balling child’s size rather using objective methods of assessment is likely to result in overweight children becoming obese before any management commences. This therefore highlights the need for further education and training for health professionals, who interact with children as a part of their professional role, in the area of paediatric growth measurement methods.

There was a belief among the participants about the need to educate the public on the benefits of routine weight status assessment and the formalisation of this practice in order for any change to occur. Education is pivotal in providing health care to families with an overweight or obese child and undertaking of routine weight status checks provides and an opportunity to determine the families’ current knowledge, and to educate on management of the child’s lifestyle. Despite this, some participants did not undertake this assessment due to a belief that families do not expect to have their child’s weight status checked or discuss weight related topics with them. Whilst a system approach to routine undertaking of this health check for primary school children would ensure all children are invited to have their weight status checked, which occurs in some countries in a school setting [8], standard consultations with health professionals should also be utilised for opportunistic weight status assessment, discussion and education.

This study has a number of strengths. Undertaking a case study approach and focusing on this regional city allowed to gain a thorough understanding of the availability of the local health services and to comprehensively map out health services offered to families in the area. This assisted with sampling, as it allowed to focus recruitment to ensure representatives from each primary health care model were sampled and there was variability in the health services and health professions, and provided an opportunity for in-depth exploration of the research question. Using one location in this study was of benefit because although the Obesity Guidelines [1] have a national reach they are implemented in a primary health care setting at a local level, and each setting determines where health professionals work and how much interaction they have with primary school aged children. Implementation of guidelines in the absence of structure results in each health professional being able to decide whether they will assess child’s weight status in their consultation or not hence focusing on one well-defined area allowed to explore the research questions at the health service and health professional level and determination of system, settings and individual level influences on the responsibility for routine weight status assessment in primary school aged children. Obtaining a relatively large and diverse sample size in a regional area of Australia provided rich and unique data about regional health professionals’ opinions. The use of COM-B and TDF frameworks provided a robust methodology and allowed for in-depth analysis. Despite these strengths, there are a number of limitations. Although there was a diverse sample size, having small number of participants per health profession and having collected limited participants’ characteristics made it impossible to investigate differences between the their responses according to their profession, age or years of practice. Further, as metropolitan areas and other countries’ may offer a different range of health services and health professionals the results from this study may not be generalizable to other areas within Australia or internationally. Sample did not include other members of the primary health care setting teams, such as practice managers or receptionists, as the focus was intentionally on primary health care professionals listed as intended audience of the Obesity Guidelines. However, non-professionals’ opinions about how weight status assessments could be included as a part of standard care in their unique service could be important to explore. Further, this study did not ask about what standard practice or consultations consisted of for each health professionals, and did not specifically investigate current practices in assessing children’s weight status nor the awareness of or compliance with Obesity Guidelines [1] despite some health professionals providing such information. Lastly, while this study provides recommendations for BCW intervention functions and policy categories it was outside of scope to appraise them according to the APEASE criteria.

### Implication for practice

In this study, health professionals’ motivation to assess children’s weight status was the central factor for undertaking this health check or recognition of own professional role in its undertaking. Results indicate that none of the study participants viewed assessing children’s weight status or raising weight related concerns during their standard consultations as a priority. According to the COM-B system [22], Motivation is powered by both Opportunity and Capability (Fig. 2), hence if it is weak or absent it fails to positively affect the Behaviour, which in this study is assessing children’s weight status. Therefore, even though Opportunity and Capability might be present it is the Motivation component of health professionals’ behaviour that appears to determine whether in a standard consultation child’s weight status is or could be assessed and/or weight related concerns raised. Hence, in order for any change to occur in practice Motivation component of the COM-B system must be effectively addressed.

According to BCW, in order to affect Motivation in the COM-B there are a number of intervention functions and types of policies that can be addressed and these are listed in Table 2. Each of them should be appraised according to the APEASE criteria which then allows to recommend specific BCT. Although this is beyond the scope of this research some logical suggestions for what can be done to positively affect health professionals’ motivation can be made based on this study’s results, previous research and approaches to behaviour change in practice, for example “Making every contact count (MECC)” [35].

**Table 2:** Motivation intervention functions with its policy functions [24]

BCW Intervention functions to affect Motivation	Link with the BCW Policy functions
1. Education 2. Persuasion 3. Incentivisation 4. Coercion 5. Training 6. Environmental restructuring 7. Modelling 8. Enablement	Regulation^1,2,3,4,5,6,8^ Service provision^1,2,3,4,5,7,8^ Communication/marketing^1,2,3,4,7^ Guidelines^1,2,3,4,5,6,8^ Legislation^1,2,3,4,5,6,8^ Fiscal measures^3,4,5,6,8^ Environmental/social planning^6,8^

MECC approach aims to maximise contact opportunities between organisations and people to positively affect behaviour change in their health and wellbeing and has been widely and successfully used in practice in the United Kingdom in variety of settings including hospitals, community services, primary care trusts [36] and optometrists’ clinics [37]. Such approach could be used to improve implementation of “ask and assess” step of the Obesity Guidelines [1] however, as emphasised in early evaluation of the MECC [38], a tailored approach for each health service would be necessary. This would be important in the context of improving the undertaking of routine weight status assessment due to variability of resource availability (staff and equipment) in each health service; however the following strategies could be considered: (1) clear division of roles regarding who and when is responsible for undertaking of the assessment and providing feedback to parents, (2) promotion of this assessment to parents in various formats to parents and staff; (3) restructure of the physical environment in the practice (for example moving scales and stadiometer to another area); (4) education and training on the benefits of the undertaking of this check, how to conduct it and incorporate it into unrelated to weight consultations; (5) nominating a “leader” in each practice who would encourage routine undertaking of this assessment, act as a resource and a role model for others; and (6) audits and discussion about performance with feedback provision and praise during staff meetings. These suggested strategies are outlined in Table 3 together with their alignments with the BCW intervention functions and rationale.

**Table 3:** Suggested strategies for affecting motivation in a health service

Suggested strategy	Connection with BCW intervention function	Rationale
Clear division of roles regarding who and when is responsible for undertaking of the assessment and providing feedback to parents	Education Enablement	Currently Obesity Guidelines [1] do not provide sufficient nor specific details about assessing primary school children’s weight status and so it cannot be defined in behavioural terms. This makes it difficult to design interventions aiming to bring on a desired change. The National Institute for Health and Care Excellence (NICE) changed how the recommendations in guidelines on schizophrenia were written to provide behaviourally specific terms [24].
Promotion of this assessment to parents and staff in various formats (for example, via video, brochures, posters, on screens in waiting rooms, flip charts on desks, in conversation with staff) and restructure of the physical environment in the practice (for example moving scales and stadiometer to another area)	Enablement Persuasion Environmental restructuring^a^	Similar strategies were use in an intervention which used COM-B, TDF and BCW and aimed to improve implementation of the “Sepsis Six” clinical care bundle [39].
Education and training on the benefits of the undertaking of this check, how to conduct it and incorporate it into unrelated to weight consultations^b^	Education Incentivisation (Continuing Professional Development points could be awarded) Training Persuasion	Intervention for smoking cessation care for Australian Indigenous pregnant women, which was designed with the BCW, used videos (training and motivational), webinars and resources to enhance motivation of health providers [40].
Nominating a “leader” in each practice who would encourage routine undertaking of this assessment, act as a resource and a role model for others	Modelling	Demonstration of behaviour by a health professional who staff aspire to is a recommended BCT under Modelling [24]. This strategy was also previously used to promote habit formation [39].
Audits of performance with provision of feedback (praise given for improved rates of weight status assessment), discussion about practice performance goals and challenges to assessment undertaking during staff meetings to generate solutions	Coercion Incentivisation (praise-social reward) Enablement (problem solving^c^) Persuasion (feedback)	Similar strategies were use in an intervention which used COM-B, TDF and BCW and aimed to improve implementation of the “Sepsis Six” clinical care bundle [39].

^a^also addresses Physical Opportunity and Physical Capability; ^b^also addresses Psychological Capability; ^c^also addresses Psychological Capability and Social Opportunity

While it may not be possible to address all of the components of Motivation to assess children’s weight status decision makers in each health service should consider if and how these recommendation could be implemented in practice.

## Conclusion

This study is the first to investigate the responsibility for routine weight status assessment in primary school aged children with the use of COM-B and TDF frameworks. Challenges with regard to health professionals’ everyday practice were highlighted and these were linked with intervention functions for changing behaviour. Further work is required to investigate in detail which intervention functions, policy categories, BCTs would be most effective, appropriate and cost-effective. These should then be piloted in practice to provide practical and evidence-based solutions that work in a real-world setting.

With the lack of clarity in Australia around who is or could be responsible for the undertaking of this check current and future opportunities for early identification of chronic positive energy balance are likely to be missed. System changes are unlikely to rapidly take place hence strategies aimed at improving the implementation of the first step in the obesity management pathway should be made at the health setting, health service and individual level. Currently, there is an opportunity to undertake this check in a general practice setting and those health professionals who work within this setting, GPs and nurses, were recognised as those having a role in assessing children’s weight status. Barriers to undertaking of this check by these health professionals should be minimised and their motivation to routinely assess children’s weight status positively affected. All primary health settings should engage those who access their services in conversations about health checks to promote routine weight status assessments in children. Currently available resource should be used more efficiently and effectively to include routine weight status assessment in standard consultations so that children who need help can commence management. The consequences of failure to identify and address childhood overweight and obesity before it reaches an extreme level are likely to have a negative impact not only on the Australian health system but most importantly on the quality of life of children and their families.

## Data Availability

The datasets generated during and analysed during the current study are not publically available due to confidentiality requirements but are available from the corresponding author on reasonable request.

## Abbreviations

APEASE: Affordability, practicability, effectiveness and cost-effectiveness, acceptability, side-effects/safety and equity
BCT: Behavioural Change Technique
BCW: Behavioural Change Wheel
BMI: Body Mass Index
COM-B: Capability, Opportunity, Motivation, Behaviour system
GP: General practitioner
MECC: Making Every Contact Count
NHMRC: National Health and Medical Research Council
TDF: Theoretical Domains Framework

## References

[1] National Health and Medical Research Council. Clinical Practice Guidelines for the Management of Overweight and Obesity for Adults, Adolescents and Children in Australia. https://www.nhmrc.gov.au/_files_nhmrc/publications/attachments/n57_obesity_guidelines_131204_0.pdf. Accessed 10 Mar 2015.

[2] Centers for Disease Control and Prevention. Developmental monitoring and screening https://www.cdc.gov/ncbddd/childdevelopment/screening.html. Accessed 15 Dec 2016.

[3] Timpka Toomas, Angbratt Marianne, Bolme Per, Hermansson Göran, Häger Anders, Valter Lars (2007). A High-Precision Protocol for Identification of Preschool Children at Risk for Persisting Obesity. PLoS ONE.

[4] Cretikos Michelle A., Valenti Lisa, Britt Helena C., Baur Louise A. (2008). General Practice Management of Overweight and Obesity in Children and Adolescents in Australia. Medical Care.

[5] Gerner Bibi, McCallum Zoe, Sheehan Jane, Harris Claire, Wake Melissa (2006). Are general practitioners equipped to detect child overweight/obesity? Survey and audit. Journal of Paediatrics and Child Health.

[6] MCFARLANE Julie, SCOTT Hayley, ROBERTSON Val, GLEESON Catherine, VANDERKROFT Dawn, WILSON Kate (2009). General practitioner and paediatrician self-reported capacity for the diagnosis and management of childhood and adolescent overweight and obesity. Nutrition & Dietetics.

[7] Sivertsen LM, Woolfenden SR, Woodhead HJ, Lewis D. Diagnosis and management of childhood obesity: a survey of general practitioners in south West Sydney. J Paediatr Child Health. 2008. 10.1111/j.1440-1754.2008.01370.x. 10.1111/j.1440-1754.2008.01370.x 18717774

[8] Davidson Kamila, Vidgen Helen, Denney-Wilson Elizabeth, Daniels Lynne (2018). How is children’s weight status assessed for early identification of overweight and obesity? – Narrative review of programs for weight status assessment. Journal of Child Health Care.

[9] World Health Organization. Declaration of Alma-Ata. www.who.int/publications/almaata_declaration_en.pdf. Accessed 16 May 2015.

[10] The Department of Health. Australian Government. Primary Health Care in Australia. http://www.health.gov.au/internet/publications/publishing.nsf/Content/NPHC-Strategic-Framework~phc-australia. Accessed 26 May 2018.

[11] Australian Institute of Health and Welfare (2014). *The measurement of patient experience in non-GP primary health care setting*.

[12] Australian Institute of Health and Welfare. Australian Government. Australia's health. Australia's health series no. 15. Cat. no. AUS 199. https://www.aihw.gov.au/getmedia/9844cefb-7745-4dd8-9ee2-f4d1c3d6a727/19787-AH16.pdf.aspx. Accessed 26 May 2018.

[13] O'Shea B., Ladewig E. L., Kelly A., Reulbach U., O'Dowd T. (2014). Weighing children; parents agree, but GPs conflicted. Archives of Disease in Childhood.

[14] Piccinini-Vallis H. (2011). Diagnosis and management of obesity: A survey of general practitioners' awareness of and familiarity with the 2006 canadian clinical Practice Guidelines. Canadian Journal of Diabetes.

[15] Australian Bureau of Statistics. 4364.0.55.001 - National Health Survey: First Results, 2014-15. Children's risk factors. http://www.abs.gov.au/ausstats/abs@.nsf/Lookup/by%20Subject/4364.0.55.001~2014-15~Main%20Features~Children's%20risk%20factors~31. Accessed 15 Dec 2015.

[16] King LA, Loss JHM, Wilkenfeld RL, Pagnini DL, Booth ML, Booth SL (2007). Australian GPs' perceptions about child and adolescent overweight and obesity the weight of opinion study. Br J Gen Pract.

[17] Davidson K, Vidgen H, Denney-Wilson E. Parental opinions about the responsibility for assessing children's weight status - a survey of Rockhampton parents. Aust N Z J Public Health. 2019. 10.1111/1753-6405.12928. 10.1111/1753-6405.12928 31390123

[18] Australian Bureau of Statistics. 2016 Census QuickStats. Rockhampton (R). http://www.censusdata.abs.gov.au/census_services/getproduct/census/2016/quickstat/LGA36370?opendocument. Accessed 20 Apr 2018.

[19] Central Queensland Hospital and Health Service. Central Queensland Medicare Local. Central Queensland Health Needs Assessment 2014. http://www.cqhealthhub.qld.gov.au/sites/default/files/CQLD%20HNA%2011Apr2014_Final.pdf. Accessed 20 Jan 2016.

[20] Australian Bureau of Statistics. 4364.0.55.001 - National Health Survey: First Results, 2014-15. Data Cubes. Table 22: Queensland. https://www.abs.gov.au/AUSSTATS/abs@.nsf/DetailsPage/4364.0.55.0012014-15?OpenDocument. Accessed 7 Mar 2019.

[21] Health Direct Australia. National Health Services Directory. https://about.healthdirect.gov.au/nhsd. Accessed 20 Apr 2018.

[22] Michie S, van Stralen M, West R. The behaviour change wheel: a new method for characterising and designing behaviour change interventions. Implement Sci. 2011. 10.1186/1748-5908-6-42. 10.1186/1748-5908-6-42 PMC3096582 21513547

[23] Cane J, O'Connor D, Michie S. Validation of the theoretical domains framework for use in behaviour change and implementation research. Implement Sci. 2012;7:37. 10.1186/1748-5908-7-37. 10.1186/1748-5908-7-37 PMC3483008 22530986

[24] Michie S, Atkins L, West R, The behaviour change wheel: a guide to designing interventions. 2014.

[25] NVivo qualitative data analysis software. QSR International Pty Ltd. 2016.

[26] Alexander KE, Brijnath B, Mazza D. Barriers and enablers to delivery of the healthy kids check: an analysis informed by the theoretical domains framework and COM-B model. Implement Sci. 2014. 10.1186/1748-5908-9-60. 10.1186/1748-5908-9-60 PMC4047437 24886520

[27] Curtis K, Atkins L, Brown K. Big hearts, small hands: a focus group study exploring parental food portion behaviours. BMC Public Health. 2017. 10.1186/s12889-017-4711-z. 10.1186/s12889-017-4711-z PMC5604285 28923032

[28] Stephan AJ, Kovacs E, Phillips A, Schelling J, Ulrich SM, Grill E. Barriers and facilitators for the management of vertigo: a qualitative study with primary care providers. Implement Sci. 2018. 10.1186/s13012-018-0716-y. 10.1186/s13012-018-0716-y PMC5806383 29422076

[29] Dettori H, Elliott H, Horn J, Leong G (2009). Barriers to the management of obesity in children - a cross sectional survey of GPs. Aust Fam Physician.

[30] Flower Kori B., Perrin Eliana M., Viadro Claire I., Ammerman Alice S. (2007). Using Body Mass Index to Identify Overweight Children: Barriers and Facilitators in Primary Care. Ambulatory Pediatrics.

[31] McMeniman Erin, Moore Romayne, Yelland Michael, McClure Rod (2011). Childhood obesity: how do Australian general practitioners feel about managing this growing health problem?. Australian Journal of Primary Health.

[32] Quelly Susan B. (2014). Childhood obesity prevention: a review of school nurse perceptions and practices. Journal for Specialists in Pediatric Nursing.

[33] Walker O, Strong M, Atchinson R, Saunders J, Abbott J. A qualitative study of primary care clinicians' views of treating childhood obesity. BMC Fam Pract. 2007. 10.1186/1471-2296-8-50. 10.1186/1471-2296-8-50 PMC2008193 17767720

[34] Spurrier Nicola J, Magarey Anthea, Wong Catherine (2006). Recognition and management of childhood overweight and obesity by clinicians. Journal of Paediatrics and Child Health.

[35] NHS. Health education England. Making every contact count. https://www.makingeverycontactcount.com/. Accessed 30 May 2019.

[36] NHS. Making Every Contact Count. Examples from Practice. https://learning.wm.hee.nhs.uk/sites/default/files/mecc_examples_of_practice_1_0.pdf. Accessed 30 May 2019.

[37] NHS England. Making Every Contact Count – Warrington Pilot https://www.makingeverycontactcount.com/media/1069/mecc-report-june-2015.pdf. Accessed 30 May 2019.

[38] Nelson A., de Normanville C., Payne K., Kelly M.P. (2013). Making Every Contact Count: an evaluation. Public Health.

[39] Steinmo S, Fuller C, Stone SP, Michie S. Characterising an implementation intervention in terms of behaviour change techniques and theory: the ‘Sepsis six’ clinical care bundle. 2015. 10.1186/s13012-015-0300-7. 10.1186/s13012-015-0300-7 PMC4529730 26253306

[40] Gould GS, Bar-Zeev Y, Bovill M, Atkins L, Gruppetta M, Clarke MJ, et al. Designing an implementation intervention with the behaviour change wheel for health provider smoking cessation care for Australian indigenous pregnant women. 2017. 10.1186/s13012-017-0645-1. 10.1186/s13012-017-0645-1 PMC5602934 28915815

